# The Effect of Annotation Quality on Wear Semantic Segmentation by CNN

**DOI:** 10.3390/s24154777

**Published:** 2024-07-23

**Authors:** Mühenad Bilal, Ranadheer Podishetti, Leonid Koval, Mahmoud A. Gaafar, Daniel Grossmann, Markus Bregulla

**Affiliations:** 1 Digital Production, AImotion Bavaria, Technische Hochschule Ingolstadt, 85049 Ingolstadt, Germanydaniel.grossmann@thi.de (D.G.); markus.bregulla@thi.de (M.B.); 2 Department of Physics, Faculty of Science, Menoufia University, Menoufia 32511, Egypt; 3 Institute of Optical and Electronic Materials, Hamburg University of Technology, 21073 Hamburg, Germany

**Keywords:** semantic segmentation, wear detection, machining tools, image annotation, U-Net model, domain expertise, labeling quality, IoU metrics, neural network performance, annotation protocols, annotation quality

## Abstract

In this work, we investigate the impact of annotation quality and domain expertise on the performance of Convolutional Neural Networks (CNNs) for semantic segmentation of wear on titanium nitride (TiN) and titanium carbonitride (TiCN) coated end mills. Using an innovative measurement system and customized CNN architecture, we found that domain expertise significantly affects model performance. Annotator 1 achieved maximum mIoU scores of 0.8153 for abnormal wear and 0.7120 for normal wear on TiN datasets, whereas Annotator 3 with the lowest expertise achieved significantly lower scores. Sensitivity to annotation inconsistencies and model hyperparameters were examined, revealing that models for TiCN datasets showed a higher coefficient of variation (CV) of 16.32% compared to 8.6% for TiN due to the subtle wear characteristics, highlighting the need for optimized annotation policies and high-quality images to improve wear segmentation.

## 1. Introduction

Deep convolutional neural networks (DCNN) are revolutionizing visual inspection in manufacturing industries. For supervised learning, high-quality annotated datasets are crucial, as the quality of annotations significantly influences model performance [1,2,3,4]. However, many available datasets suffer from improper annotations and instance labeling errors, adversely impacting the performance of learning algorithms [5,6].

Restricting annotations to a single label, similar to ImageNet, can result in inaccuracies because images might encompass multiple objects, which in turn may contain sub-objects or different classes. Furthermore, encouraging users to annotate images that should not be included in the dataset introduces inconsistencies and biases [7,8]. Growing skepticism surrounding datasets derived from user-generated content on the Internet has led to discontinuation or revision of several popular benchmarks. The ongoing use and distribution of these datasets in the form of duplicates or subsets also raise concerns. AI-based quality control in manufacturing faces similar challenges [9]. A survey [10] that interviewed 227 participants from five continents and 20 different industries found that 76% of the participants agree that training data quality and its labeling represent significant challenges in AI projects. To get AI systems off the ground, training data must be extensive and precisely labeled and commented. The use of AI is becoming an increasing priority for companies. Data scientists are under pressure to deliver projects but often need to provide training data of the required size and quality. In the manufacturing sector, the challenges extend beyond data aggregation to dataset selection and labeling, introducing potential biases. In object recognition tasks, even ensuring accurate and consistent placement of bounding boxes around objects is challenging in terms of labeling quality. The authors of [11] observed that sketching a bounding box is both more intricate and time-consuming than annotating classification labels, typically achieved via multiple-choice queries. The author showed that labeling through a suitable process and guideline, in several steps from quality control and training the performance model, can be significantly improved. Therefore, it can be strongly expected that the task of annotating different types of wear presents a higher number of challenges. Tool wear is divided into two main categories: normal wear and abnormal wear. Normal wear occurs as a normal consequence of machining and is influenced by factors such as cutting parameters, tool material and workpiece material [12,13]. However, abnormal wear occurs due to unfavorable reasons such as excessive cutting forces, poor lubrication, material adhesion or complete tool failure. The identification of abnormal wear can be used to take appropriate measures to optimize the geometric parameters of a tool and to ensure the quality of the workpieces.

In order to distinguish between normal and abnormal wear, the neural network must be fed a dataset with consistent annotation to differentiate between the different wear categories for semantic segmentation. In the standard semantic segmentation approach, including our CNN architecture, each pixel is assigned to a single category (e.g., normal wear, abnormal wear, tool).

In this paper, in addition to the deep approach of wear detection, we will also analyze in detail the difficulties that arise in labeling with respect to the normal and abnormal wear of geometrically complex cutting tools by using our CNN systematically.

To the best of our knowledge, there is no previous work that specifically addresses this problem of annotating datasets for wear detection and systematically addresses the effects of different annotations on the performance of the model. Here, we clearly show the challenges in wear detection reduction and at the same time the role of expertise in developing an AI-based wear detection model with respect to the four classes: “Abnormal Wear”, “Normal Wear”, “Tool” and “Background”. This is only feasible due to our innovative detection system, which has already been registered and published at the European Patent Office [14]. This system enables reproducible imaging of the tool and signs of wear, thus reducing effects such as reflections on the surface and fluctuations in the light source. Otherwise, it would not be possible to compare the models from different annotators using conventional imaging methods.

The article is organized as follows: Section 2 discusses the state of the art regarding the relevance of marking quality and CNN-based approaches for AI applications and research. Section 3 introduces the methods and discusses the background and the challenge of tool manufacturers in image-based inspection of cutting tools. The focus is on their optical properties, especially their technical and geometrical parameters. Section 3 also presents the CNN-based methods and the guidelines for the detection of normal and abnormal wear. Section 4 discusses the performance of the different modelers trained by different annotators. Finally, the wear segmentation results of the different annotators with different grades of expertise are reviewed using the CV to identify the main inconsistencies during labeling and to evaluate the performance of the model on two different types of end mill datasets.

## 2. State of the Art

In this section, we consider the related studies, which cover two main areas. First, we deal with data-based AI. This includes an examination of existing annotations for benchmark datasets. We then look at new AI-based methods for wear detection. We then give a brief overview of the state of the art in artificial neural network techniques such as semantic segmentation and object recognition, where we also focus on the quality of labeling in terms of wear detection.

It is well known that the importance of data quality and preparation is of particular interest in the development of artificial intelligence models. Data-centric AI and the improvement of datasets are not equivalent. A data-centric AI competition focuses on the quality, relevance, and robustness of the data used to train AI models. Data-centric AI approaches focus mostly on systematically improving data to achieve a model with the best performance. Meanwhile, model-centric approaches focus on code or model architecture improvement to enhance performance. Both methods can be balanced well to provide a robust AI solution [15]. There has been a significant effort to improve the performance of CNN models using data-centric approaches. The authors in [16] discuss the relevance of data-centric methods for structural health monitoring and resilience.

In computer vision, most research on dataset validation has traditionally focused on validation against the image database such as [17] and the verification of associated annotations. ImageNet has been the subject of numerous studies recently. ImageNetV2, which is described in detail in [18], experienced a significant drop in performance for numerous classification models, as [18,19] shows. Labeling discrepancies were found in several prominent datasets, leading to performance degradation, especially in DCNN [5]. In a study by [20], bird specialists discovered about 4% of annotation errors in bird images from the CUB-200-2011 [21] and ImageNet datasets. In principle, biases within datasets can lead to reduced model performance. According to Esteva et al. [22], the lack of extensive, high-quality labeled datasets is a major obstacle to the use of supervised deep learning for medical imaging. Taran and colleagues [23] used the Cityscapes dataset, which contains both fine and coarse annotated ground truth data, to investigate the effects of annotation quality on the performance of semantic image segmentation in traffic conditions [24]. The authors investigated two scenarios: first, using the fine ground truth annotations for both training and inference; second, training with the fine annotations followed by inference using the coarse ground truth annotations. For the semantic segmentation model, the research group in [25] used a Pyramid Scene Parsing Network (PSPNet), and they analyzed a subset of the Cityscapes dataset, which included data from three different cities and the following classes: roads, cars, pedestrians, traffic lights, and traffic signs. The dataset intentionally contains 20,000 additional images with coarse annotations to support methods that utilize large amounts of weakly annotated data. The authors used average IoU as a metric. In their results, they found that IoU values for training data using both fine and coarse labeled data were generally higher than those for images with fine ground truth. Based on the results of these comparisons between fine and coarse ground truth annotations, the authors suggested that deep neural networks could be used to generate datasets with coarse ground truth. These could then be modified and used to fine-tune pre-trained models for specific applications.

Currently, to our knowledge, there is no research on the influence of annotation quality and skill level of a worker on model performance for identifying various wear types of cutting tools, despite the high demand for AI-based tool wear inspection systems. Recent research in tool condition monitoring (TCM) has widely been focused on wear detection using various CNN architectures such as [26,27]. Employing the automatic convolutional encoder (CAE), Xuefeng Wu and colleagues adapted a network model specifically for wear detection, refining model parameters through the backpropagation method in tandem with the stochastic gradient descent (SGD) algorithm [26]. In a parallel vein, Thomas Bergs and team employed the Fully Convolutional Network (FCN) and U-Net for the semantic segmentation of individual tool datasets. Their objective was to identify wear on a microscopic scale. For the labeling process, both [26,27] use a standard direct light source to generate high-resolution images from optical microscopy for tool wear labeling and inspection. In contrast, our approach ensures proper illumination of the tool to obtain high-quality images without reflections from the entire tool. While the referenced methods use a microscope and capture images under a single lighting condition, making it difficult to inspect the entire tool and detect wear in different shapes and forms, our approach overcomes these limitations by eliminating illumination artifacts and thereby improving wear detection. The results for a limited tool scene, due to changes such as light exposure, yield a mean IoU coefficient of 0.73 [28]. However, due to the complicated geometry and structure of the tools, capturing suitable images for advanced AI applications remains a challenge [29].

U-Net [30] and Mask R-CNN [31] are two of the leading deep learning frameworks known for their superior performance in instance segmentation. Both achieved remarkable results during the 2018 Kaggle Data Science Bowl [32,33]. Mask R-CNN utilizes multiscale feature maps to capture robust semantic features, with the aim of effectively delineating the boundaries of the cervical nuclei [34]. However, Mask R-CNN demands significant computational power, rendering it less suitable for large-scale classification tasks in industrial inspections.

In the context of semantic segmentation, the U-Net has demonstrated superior performance compared to conventional convolutional networks by using the sliding window approach. U-Net architecture has been used for various applications such as medical and biomedical imaging and remote sensing image analysis [35,36]. This method entails applying a fixed-size window across different segments of the input image to discern features. U-Net’s distinctive “U-shaped” architecture enables it to adeptly capture context and precisely localize it, presenting a more efficient strategy than the sliding-window technique. U-Net’s architecture comprises encoder blocks and decoder blocks. The encoder blocks condense the input image to discern features across varied scales via convolutional layers. Max-pooling operations further diminish the spatial dimensions of these feature maps. In contrast, the decoder blocks are tasked with upsampling the feature maps. Transposed convolutions are used for this upsampling process to recapture spatial information that was previously lost during the downsampling phase. Skip connections are integrated to merge the feature maps from both encoder and decoder blocks, ensuring the network preserves crucial details during the upsampling process. For our analysis, inspired by the U-Net presented in [30], we developed a CNN architecture with three encoding and decoding blocks utilized for images with three channels (RGB) to maintain visual information. The bottleneck layer between the encoding and the decoding blocks represents the latent space that holds the most compressed representation of the training dataset.

## 3. Materials and Methods

### 3.1. Structure Parameter-Related Annotation Challenges

In this section, we discuss the challenges associated with annotating images from integral spiral cutters, focusing on their pivotal technical and geometric parameters. We then introduce the innovative Image Acquisition System (IAS), designed to capture no-reflection images of tools, ensuring optimal visibility of wear for annotation purposes.

**Figure 1:**
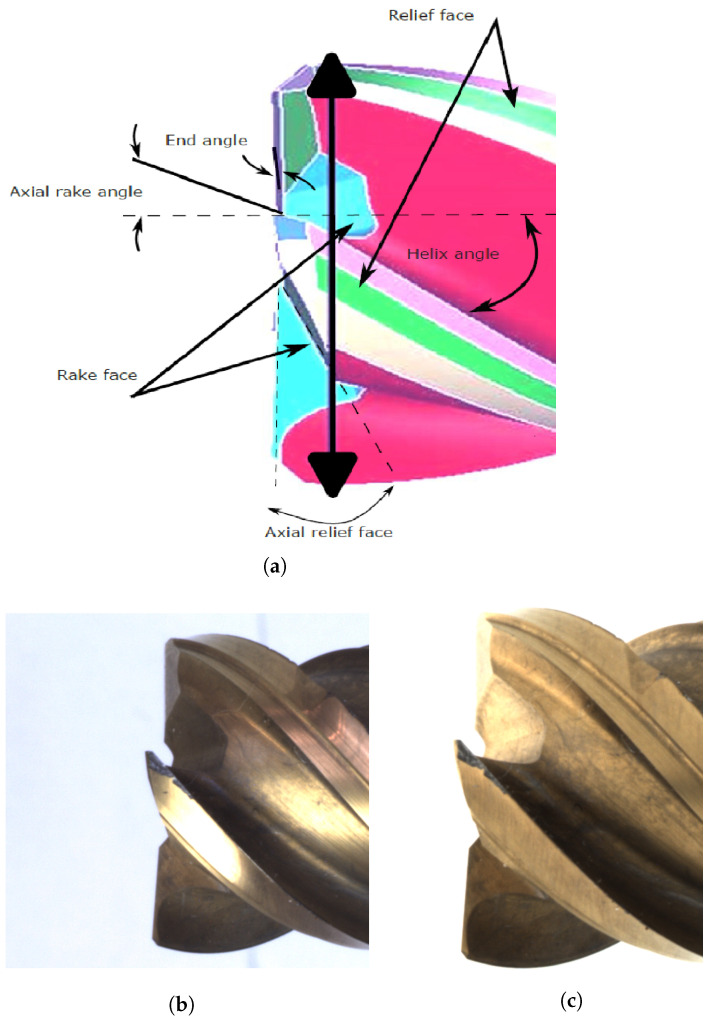
(**a**) Important geometric parameters of an end mill, such as relief face, end angle on the axial rake, rake face, axial relief face, and helix angle. (**b**) Illustration of light reflection on a TiN-coated end mill when illuminated by standard direct diffuse lighting. Notably, the most intense reflection is observed along the cutter’s edges, while shadowing is evident within the inner rake space. (**c**) Image captured by the IAS.

Integral spiral milling cutters are routinely used in milling processes to machine complicated workpieces made of different materials. The geometry of the tool is crucial for the efficiency and quality of the milling process. Different geometric properties of the cutting edges have a major influence on the overall quality of the milled product. Figure 1a,b show the complexity of describing the wear characteristics of these tools, while taking into account their specific geometric parameters and the resulting reflection behavior on the end mill surface, which makes the annotation process difficult. We solve this problem by capturing images with IAS (Section 3.2), which avoid reflection in imaging the tools, as shown in Figure 1c.

In addition to the optical properties, the complex geometry of these tools makes it difficult to identify wear patterns and even more difficult to differentiate between normal and abnormal wear. Fluctuating light conditions further complicate the detection and differentiation of signs of wear [37]. Consequently, such complex-shaped and optically critical tools provide an ideal way to investigate the role of expertise in providing labeled datasets and its impact on the quality of the annotations when evaluating the performance of different models.

### 3.2. Acquisition System

**Figure 2:**
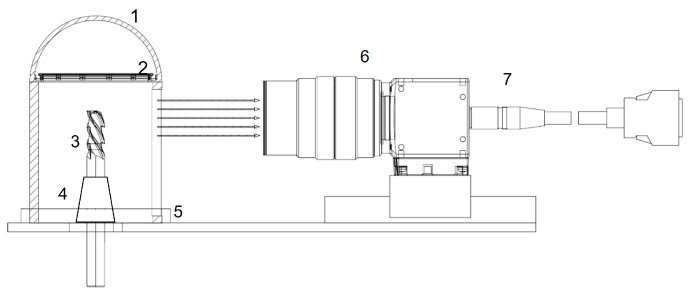
Schematic representation of the measurement setup for capturing high-quality images of end mills. The acquisition system consists of (**1**) a hemisphere with barium sulfate coating, (**2**) 12 LEDs located at the edge of the hemisphere, (**3**) a tool to be examined, which is held by (**4**) a three-jaw chuck, (**5**) represents the rotating plate for a 360° recording, (**6**) is a camera with an interface (**7**) connected to the computer.

To ensure high reproducibility when capturing images of end mills, we utilized the acquisition system depicted in Figure 2. The system employs a centrally-aligned three-jaw clamping chuck (4) to secure the tool (3) precisely at the center of the housing cavity (1). An LED ring, positioned on the inner surface of the hemisphere, disperses the electromagnetic radiation emitted from the emitter uniformly across the object. The housing’s (1) design includes a radially symmetrical segment, further promoting multidirectional light scattering. This layout allows for the electromagnetic radiation to undergo multiple reflections within a U-shaped region, thereby amplifying the diffusion effect produced by the diffuser.

For a complete tool inspection, a motorized rotating plate (5) holds the three-jaw chuck. This arrangement allows for the continuous capture of the end mill from various angles using a single camera. While parts of the structure reflect the electromagnetic radiation, they incorporate openings or transparent sections, enabling the radiation to traverse from the interior to the camera system (6) and interface for data transfer of the images (7). The images were taken with a commercial Nikon camera (Nikon D800E, Nikon Corporation, Tokyo, Japan), with a 105 mm lens. The tool was a four-edged end mill (106.5 mm in length, 40 mm in flute length, 15.4 mm in diameter, 16 mm in shank diameter). For the TiCN dataset, an aperture of f/29 was used with a similar four-edged end mill (93 mm length, 37 mm flute length, 16 mm diameter, 16 mm shank diameter). Both sets of data were taken in equidistant angular steps of 15° from 0° to 360° with white illumination. The field of view of the 105 mm lens on a full-frame sensor is approximately 23.3 degrees diagonal. Smaller apertures such as f/32 and f/29 provide a large depth of field, which is beneficial for capturing detailed images of tool wear. The images have been cropped to focus on the tool and minimize background noise. The Nikon D800E’s pixel pitch of 4.87 microns ensures that each pixel corresponds to approximately 4.87 microns on the tool surface. Given the importance of high-resolution images for effective model training and enhanced wear and damage detection, it is essential to capture high-quality images with great resolution. Consequently, high-resolution images were segmented into 32 discrete smaller images. This not only bolsters model training but also refines the detection of wear and damage on a microscopic scale.

### 3.3. Annotation Guideline

**Figure 3:**
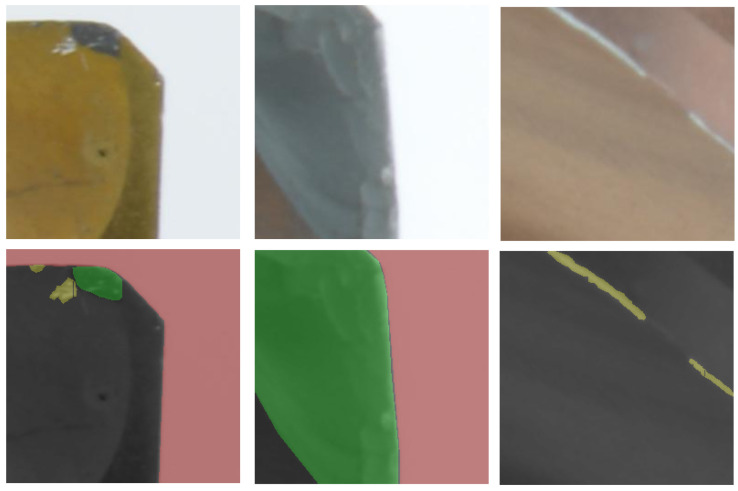
Positive annotation examples. The wear classification includes two primary categories: “yellow” represents typical wear and “green” denotes abnormal wear and the two additional categories: “red” for the background and “black” for the tool.

**Figure 4:**
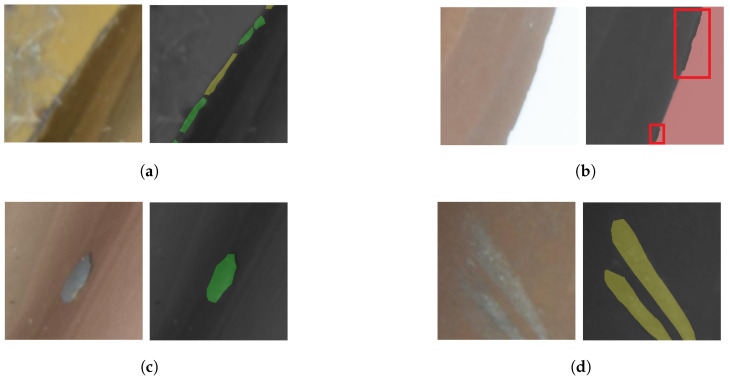
Negative examples of annotations. Each sub-figure highlights a distinct type of incorrect annotation. The classifications include: “yellow” for normal wear, “green” for abnormal wear, “red” for the background, and “black” for the tool itself. (**a**) shows mislabeling abnormal wear as normal wear, (**b**) incorrect annotations mistakenly marking the background as a part of the tool surface, (**c**) impurities have been labeled as abnormal wear, (**d**) material removal due to chip residue at chipping space has been marked as normal wear.

The annotation of our dataset was carried out by three annotators, each having varying levels of experience in machining. They all commenced their annotation tasks concurrently. Annotation was performed on the full images. We divided the images into 32 small fragments. Prior to the main annotation task, each was required to undergo training on a predefined dataset, ensuring their annotations aligned with established ground truths. They were also provided with examples of both normal and abnormal wear conditions to achieve a consistent annotation baseline. To ensure high-quality annotations, we formulated detailed instructions for the annotations:

1. **Definition:** *Normal wear* is characterized by wear without fractures. In contrast, *abnormal wear* signifies wear with fractures. Both types of wear are considered contiguous surfaces.
2. **Positive Examples (refer to Figure 3):**
3. **Negative Examples (please see Figure 4):**
  (a) Mislabeling abnormal wear as normal wear (Figure 4a)
  (b) Annotations mistakenly marking the background as a part of the tool (Figure 4b)
  (c) Incorrect annotations marking impurities as abnormal wear (Figure 4c)
  (d) Misidentifying worn regions within the chipping space as normal wear (Figure 4d)
4. **Additional Guidelines:**
  (a) Only label damage present on the cutting edges or phase, excluding the chipping space.
  (b) Wear that is ambiguous and cannot be distinctly labeled should be excluded from the dataset.
  (c) Instances can appear overlapped, but in effect, they do have finer boundaries that can merge into one another, especially at the cutting edges. Here, careful annotation is required.

Before the annotators started working on a new dataset, they performed a trial run in which they selected 10 difficult image examples from the dataset. The dataset of two tool types with two different coatings contains four instances assigned to four target classes: normal wear conditions, abnormal wear conditions, tools, and background. During the annotation process, it was discovered that certain images in the original dataset did not contain clear recognizable wear patterns. These images were subsequently removed from our dataset.

### 3.4. Cnn Model

**Figure 5:**
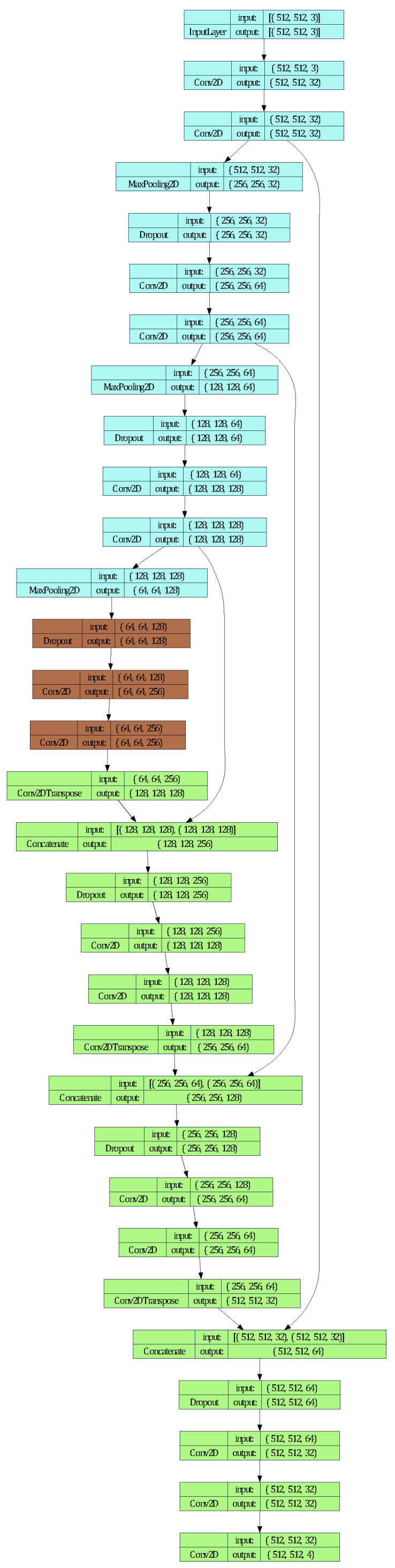
CNN architecture for normal and abnormal wear segmentation. Encoding blocks are colored in blue. The bottleneck layer is colored in brown. Decoding blocks are colored in green.

A CNN architecture with three encoding and decoding blocks has been used to train models for normal and abnormal wear detection. The detailed architecture is shown in Figure 5. The CNN architecture consists of three encoding and decoding blocks utilized for images with three channels (RGB) to maintain visual information. The bottleneck layer between the encoding and the decoding blocks represents the latent space that holds the most compressed representation of the training dataset. The training parameters have been reduced to 2,140,740 and are listed with other relevant parameters below in Table 1. BS stands for batch size, which refers to the number of training examples utilized in one iteration. DO stands for dropout rate, which is a regularization technique used to prevent overfitting in neural networks by randomly dropping units during training.

### 3.5. Dataset Characteristics

A total of 24 high-resolution images of each tool were captured in 15° angle increments for this study. Each image was finally split into 32 fragments of pixel size 512 × 512. For this purpose, only the cutting area of the tools was taken into account. Thus, a total of 768 images were generated for training and testing the neural network for each tool. The participating employees came from Linner Werkzeug Schleif Fabrik GmbH (https://herionlinner.com/linner-gmbh-werkzeugfabrik/ (accessed on 10 May 2024)), a company specializing in tool regrinding, with varying work experience ranging from 1 to 20 years. The tools have been used on CNC machines to produce gear racks by CNC finishing at WMH Herion Antriebstechnik GmbH (https://herionlinner.com/antriebstechnik/ (accessed on 10 May 2024)).

The average time taken to annotate an image was 45 min for a whole image, which is approximately 1.5 min per image fragment. Annotation was performed on the full images. The annotators used LabelMe software (v5.0.1) [38] (https://pypi.org/project/labelme/ (accessed on 10 May 2024)) to label sample images for this study. To qualify as proficient annotators, each candidate was required to complete an image annotation training program. This program consisted of three steps: tutorials on how to use the software for annotation using polygons, distinguishing different types of wear, and adhering to the guidelines outlined in Section 3.3.

We compare a carefully annotated dataset with alternative annotations created by individuals from diverse professional backgrounds. The three people did not follow the same annotation instructions. The resulting models must be able to differentiate various wear patterns in complex end mills. The annotation process involves participants with varying skill sets, ranging from novices to experts. We expect that, even when detailed guidelines are provided, annotations produced by experienced professionals will exhibit a higher level of precision and consistency compared to those produced by their less experienced colleagues. The main relevant aspects that can affect CNN model performance by the dataset are:

1. **Tool Diversity and Wear Patterns:** Our experimental framework leverages two distinct datasets to ensure a comprehensive evaluation of various wear patterns.
  - *Dataset 1:* encompasses tools coated with Titanium Nitride (TiN).
  - *Dataset 2:* incorporates tools coated with Titanium Carbonitride (TiCN).
2. **Optimizing CNN Models:** Images from the datasets were strategically resized to dimensions of 512 × 512 pixels, facilitating compatibility with our CNN model and optimizing computational performance.
3. **Data Partitioning:** The assembled images are systematically divided into training, validation, and testing segments, following a 8:1:1 distribution. A detailed enumeration of the instances in the dataset is presented in Table 2.

To obtain an estimation of the instances, the number of individual instances labeled as polygons by annotator 1 was calculated. The number of instances of Background and Tool is easy to check here. Since each annotator labels its own dataset to independently train its own model for wear detection, the number of instances and pixel sizes of normal and abnormal wear may vary for each dataset of distinct annotators.

**Figure 6:**
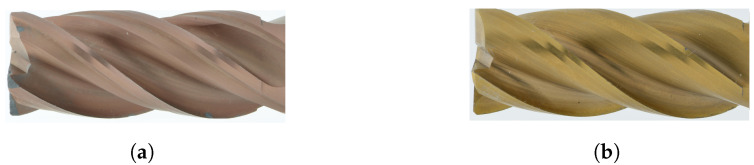
Illustrative images captured via the acquisition system: (**a**) TiCN-coated endmill and (**b**) TiN-coated endmill.

Marking wear phenomena requires a great deal of precision. Considering the time involved in generating and annotating datasets, our efforts were focused on two particular tool coatings: TiCN (Figure 6a) and TiN (Figure 6b). Apart from the differences in coating, they have different wear patterns, wear contamination, and specific applications. Both coatings have their unique strengths: TiCN, prevalent in end mills, is robust and widely used for machining steel and cast iron. On the other hand, TiN is renowned for its wear resistance and low friction coefficient, making it a popular choice for various cutting applications [39].

To expedite model training without compromising on image quality, we segmented the original images. This ensured quick training and preserved critical visual data that could otherwise be lost by compression. For our ablation study, we used the CNN architecture presented in Figure 5. Through an examination of various hyperparameters, such as Learning rate (LR), Batch size (BS), and dropout rate (DO), we determined the optimal settings for the multiple-class segmentation results.

### 3.6. Annotators

For a comprehensive evaluation of the impact of annotation quality on modeling results, we assigned several annotators with different levels of expertise to annotate each dataset.

- **Annotator 1:** with more than two decades of experience in the field, this person embodies the highest level of expertise and experienced insight into this topic.
- **Annotator 2:** with 2 years of hands-on experience, this participant represents the middle tier, bridging the gap between novices and veterans.
- **Annotator 3:** as a newcomer to the field of machining technology, this participant offered a fresh perspective without deep-rooted biases or ingrained expertise.

### 3.7. Evaluation Indicators

To evaluate and compare segmentation models, we employ the accuracy metric known as Intersection over Union (IoU). The Jaccard Index is used as a metric to investigate the similarity in pixel-wise matter between Ground Truth (GT) and prediction.

(1) $$J\left(A,B\right)=\frac{\left|A\cap B\right|}{\left|A\cup B\right|}=\frac{\left|A\cap B\right|}{\left|A|+|B|-|A\cap B\right|}$$

In this formula, J(A,B) represents the Jaccard Index between sets A and B. The numerator |A∩B| is the size (cardinality) of the intersection of sets A and B, and the denominator |A∪B| is the size of the union of sets A and B. This metric provides a measure of the overlap or similarity between the two sets, with values ranging from 0 (no overlap) to 1 (complete overlap or similarity).

To evaluate the models on their overall performance, we introduce a weight adjustment of the under-represented classes such as normal and abnormal wear compared to the over-represented classes such as background and damage-free tool surface. The formula to determine the weights for wmIoU are described below:

1. Determine the class frequencies by counting the occurrences of each class in the dataset to obtain $N_{1},N_{2},N_{3},$ and $N_{4}$.
2. Calculate the inverse frequencies for each class as follows:
  (2) $$\frac{1}{N_{1}},\frac{1}{N_{2}},\frac{1}{N_{3}},\;\mathrm{and}\;\frac{1}{N_{4}}.$$
3. Normalize the weights by summing all the inverse frequencies and then divide each inverse frequency by this sum to obtain weights $w_{1},w_{2},w_{3},$ and $w_{4}$ that add up to 1:
  (3) $$w_{i}=\frac{\frac{1}{N_{i}}}{\sum_{j=1}^{4}\frac{1}{N_{j}}}$$
4. Apply the weights to calculate the weighted mean IoU:
  (4) $$\mathrm{wmIoU}=w_{1}\cdot IoU_{1}+w_{2}\cdot IoU_{2}+w_{3}\cdot IoU_{3}+w_{4}\cdot IoU_{4}$$

Using inverse frequencies, we ensure that underrepresented classes (with a lower frequency $N_{j}$) are given more weight in the calculation. This increases the influence of the underrepresented class on the average performance evaluation of the model. In addition to our analysis, we used the CV to assess the relative variability of the mIoU model performance of the three annotators. We use the CV as a standardized metric of dispersion that is particularly useful for comparing the degree of variation in prediction performance of the models that have been trained on different labeled datasets. The formula for calculating the coefficient of variation is as follows:

(5) $$CV_{IoU_{i}}=\frac{\sigma_{IoU_{i}j}}{\mu_{IoU_{i}j}}\times 100\%$$

where σ represents the standard deviation and μ the mean of the segmentation results for the class *i* of all annotators *j* = 1, 2, 3.

## 4. Results and Discussion

In this research paper, we address the following main goals. First, we investigate the challenge of annotating wear of different types to improve our annotation guidelines for wear segmentation in its different forms. Second, we compare the performance of the different models from the different annotators and investigate the impact of annotation quality on the performance of our proposed CNN models. Then we verify the impact of the hyperparameters with respect to both datasets—TiN and TiCN—which have been annotated by different annotators. Here, we investigate which of the hyperparameter combinations are particularly sensitive to varying annotation quality.

### 4.1. Comparison of Annotation by Different Annotators

**Figure 7:**
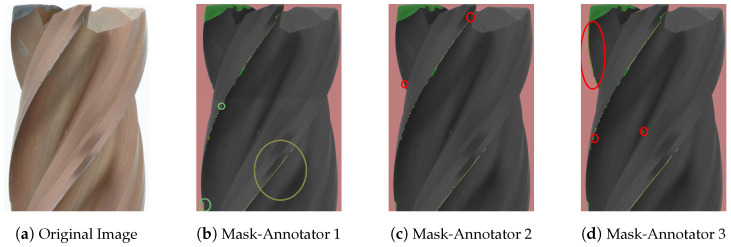
Masks of endmill wear annotations for comparison of a TiCN-coated endmill dataset. The annotations were performed by three annotators: Annotator 1, Annotator 2, and Annotator 3. The dataset includes four classes: normal wear in green, abnormal wear in yellow, background in red, and tool in black. The critical annotations have been marked in red.

**Figure 8:**
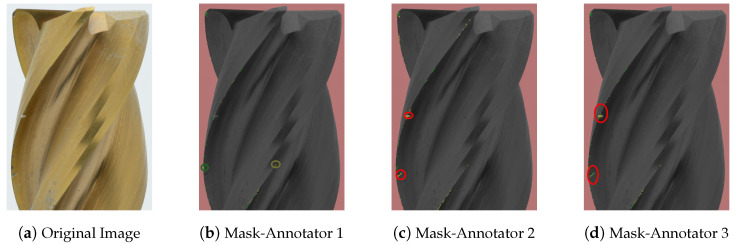
Masks of Endmill wear annotations for comparison of a TiN-coated endmill dataset. The annotations were performed by three annotators: Annotator 1, Annotator 2, and Annotator 3. The dataset includes four classes: normal wear in green, abnormal wear in yellow, background in red, and tool in black. The critical annotations have been marked in red.

A comparative analysis of the annotations revealed interesting patterns. Although the annotations for the categories “tools” and “background” show good agreement, there is a noticeable variability in the labeling of “normal” and “abnormal” wear. This variability can be clearly seen in Figure 7 and Figure 8. The critical annotations have been marked in red.

The reasons for these discrepancies are complex:

1. Ambiguity in wear assessment: in particular, minute wear features on cutting edges, such as on the edge of the TiCN cutter, presented a challenge in definitive categorization, but still shows consistency in annotation (marked green and yellow in Figure 7b).
2. Concentration loss: as can be seen in Figure 7b,c, noticeable wear patterns (marked in red at the top left part of Figure 7d) were occasionally missed. This oversight could be due to diminishing concentration during the annotation process.

Given these findings, it is of crucial importance that we include an additional control level in the annotation process workflow. We propose an additional annotation check aimed at preemptively identifying obvious inconsistencies in the annotation and taking measures at an early stage.

### 4.2. Performance Comparison of Various CNN Models on Diverse Datasets from Multiple Annotators

In this subsection, we present a detailed evaluation of the performance of our proposed CNN model by varying hyperparameter combinations. The models have been trained on both datasets, i.e., the TiN- and TiCN-coated ones. Each training dataset has been labeled by one of the annotators, resulting in a total of six annotated datasets. We evaluate the adaptability and performance of these wear inspection methods with respect to the labeling quality and identify subtleties that arise from different data, hyperparameters, and different annotations. All of these factors can affect the performance of a model. We will investigate the factors that influence performance and conclude with potential strategies to improve the robustness and generalization of models for wear detection tasks.

To examine the role of labeling in affecting the effect of annotation quality, Table A1 and Table A2 present the mean IoU in TiN and TiCN inference data predicted by models trained on different datasets labeled by annotators 1, 2, and 3. We consider the mIoU of the distinct classes: “Abnormal Wear”, “Normal Wear”, “Tool”, and “Background”, as well as the overall performance wmIoU. The models have been trained with different hyperparameters. The LR has been set to 0.001 and 0.0001. We also varied the DO rate for each layer: 0.0, 0.3, 0.5, and experimented with different BS: 8 and 16. We employed the sparse categorical cross-entropy loss function to train the model for all combinations of parameters.

It can be seen from Table A1 and Table A2 that all annotators achieve remarkable results for the class “Background”, with a high mIoU of 0.99 for this class in both datasets, coated with TiN and TiCN. Taking into account the class “Tool”, the annotators performed better on the TiN dataset (Table A1) compared to the TiCN dataset (Table A2).

While Annotators 1, 2, and 3 achieved a higher mIoU of nearly 1.0 for multiple models (except for Annotator 2) for the class “Tool” on the TiN dataset, the models from annotators predicted on the TiCN-coated dataset achieved for the class “Tool” a maximum mIoU of 0.96 for Annotator 1 (A1MTiCN 3, LR: 0.001, BS: 16, DO: 0) and 0.94 for Annotator 2 (A2MTiCN 2, LR: 0.001, BS: 8, DO: 0.5). The model A3MTiCN 1 (LR: 0.001, BS: 8, DO: 0.3) of Annotator 3 achieved a maximum mIoU of 0.97. This suggests that the complexity of tool features, possibly combined with variations in annotator labeling, affects model performance, even for the class “Tool”.

For research interest, the classes “Normal Wear” and “Abnormal Wear” are mostly relevant since their labeling quality and impact on performance can be dependent on the level of expertise the annotators have. Regarding normal and abnormal wear, the TiN-coated milling tool compared to the TiCN-coated milling tool achieved a significantly higher mIoU value for almost all combinations of hyperparameters, as seen in Table A1. The model A1MTiN 1 (LR: 0.001, BS: 8, DO: 0.3) of Annotator 1 achieved a remarkable mIoU of 0.82 on the TiN-coated dataset for the class “Abnormal Wear”, and for the class “Normal Wear”, an mIoU of 0.71. While model A2MTiN 6 (LR: 0.0001, BS: 8, DO: 0.0) from Annotator 2 achieved similar high performance with an mIoU of 0.81 for abnormal wear but only 0.46 for normal. The best model trained on the Annotator 3 dataset is A3MTiN 1 (LR: 0.001, BS: 8, DO: 0.3), achieving a maximum mIoU of 0.75 for the class “Abnormal Wear” but only a poor mIoU of 0.57 for the class “Normal Wear”.

For comparison, ref. [40] achieved the highest score of 0.55 with LinkNet for flank wear (normal wear), and for the class groove (abnormal wear), achieved the highest score of 0.80 with U-Net. It must be mentioned that these results [40] stem from optical microscopic images that consider only a small region of the tool. In our case, our results come from the entire tool itself.

Considering the TiCN-coated endmill, the overall IoU results are rather poor, as can be seen in Table A2 for abnormal wear and for normal wear classes.

Annotator 1 achieved the best wear segmentation results of mIoU = 0.66 for abnormal wear and 0.59 for normal wear with the hyperparameter combination A1MTiCN 1 (LR: 0.001, BS: 8, DO: 0.3). Annotator 2, in model A2MTiCN 2 (LR: 0.001, BS: 8, DO: 0.5), achieved the best IoU segmentation results of 0.60 for abnormal wear and 0.56 for normal wear. Although Annotator 3’s performance in A3MTiCN 2 (LR: 0.001, BS: 8, DO: 0.5) for abnormal wear segmentation was better than Annotator 2, the segmentation results for normal wear were poorer, with an mIoU of 0.40.

Regarding the hyperparameter tuning, we can observe that models with adjusted DO, in particular those at 0.3 and 0.5, tend to deliver the best performance, suggesting that regularization via dropout could be impacting the model’s ability to generalize from training data.

For generalization, the use of dropout layers as a regularization method, especially with a DO of 0.3, generally seems to improve the wmIoU across all annotators. A higher BS = 16 also appears to result in a slightly lower wmIoU for all models and datasets compared to a smaller BS = 8. In comparison, wmIoU values tend to perform better with LR = 0.001 than with LR = 0.0001.

### 4.3. Impact of Hyperparameters on Model Sensitivity to Annotation Quality

**Figure 9:**
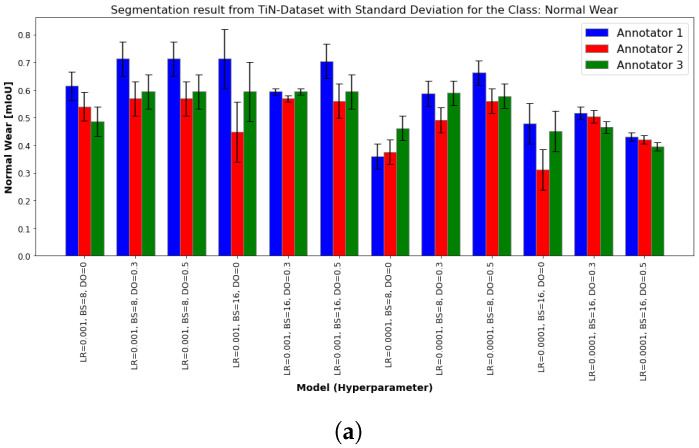

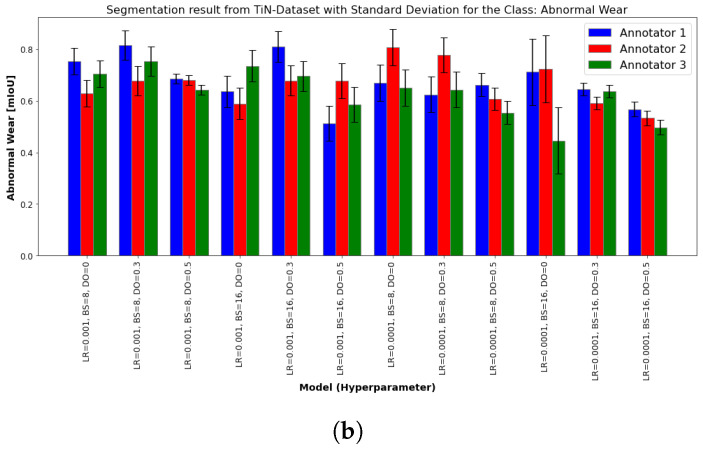
mIoU results of various models for classes of interest: (**a**) normal wear and (**b**) abnormal wear. These models were trained using the same dataset but labeled by different annotators. The LR was set to 0.001 and 0.0001, and hyperparameters such as BS and LR varied, as detailed in Table A1. The dataset originates from a TiN-coated end mill. The standard deviation is depicted to illustrate the performance variation among annotators 1, 2, and 3.

**Figure 10:**
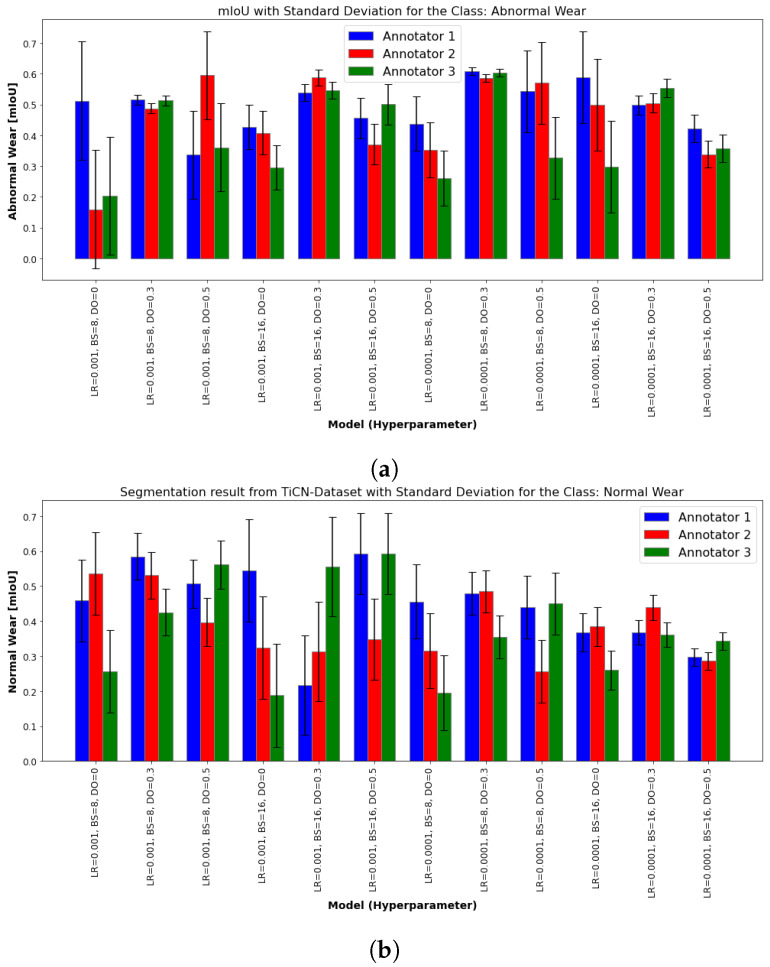
mIoU results of various models for classes of interest: (**a**) normal wear and (**b**) abnormal wear. These models were trained using the same dataset but labeled by different annotators. The LR was set to 0.001 and 0.0001, and hyperparameters such as BS and LR varied, as detailed in Table A2. The dataset originates from a TiCN-coated end mill. The standard deviation is depicted to illustrate the performance variation among annotators 1, 2, and 3.

To consider the differences and sensitivity of the model with regard to possible annotation errors, the figures below present the segmentation results for “Normal Wear” and “Abnormal Wear” classes from the TiN (Figure 9) and and TiCN (Figure 10) datasets, evaluated using the mIoU and the standard deviation between the annotators. Each figure compares the performance of various models, each defined by specific hyperparameters: LR, BS, and DO. The results are evaluated by three different annotators and the variability between their annotations is shown through standard deviation error bars.

The presence of a higher standard deviation in some models suggests that these models are more sensitive to annotation differences. It can be observed that certain hyperparameters can make a model more sensible to incorrect annotations. This sensitivity means that the performance of the model can vary significantly depending on the quality and consistency of the annotations. The mIou results of normal and abnormal wear of the TiN tool (Figure 9a,b) and normal wear (Figure 10b) of the TiCN tool show a similar trend where the models with LR = 0.001 outperform the models with LR = 0.0001. The model with hyperparameters LR = 0.0001, BS = 16, and DO = 0 shows greater performance variability between the annotators in the class, as shown by the larger standard deviation error bars. This trend cannot be observed for the abnormal wear of TiCN in Figure 10b. In general, models with dropout rates of 0.3 and 0.5 tend to perform better. Annotator 1 consistently yields higher mIoU values, but there is obvious variability between the annotators.

### 4.4. Visual Analysis

**Figure 11:**
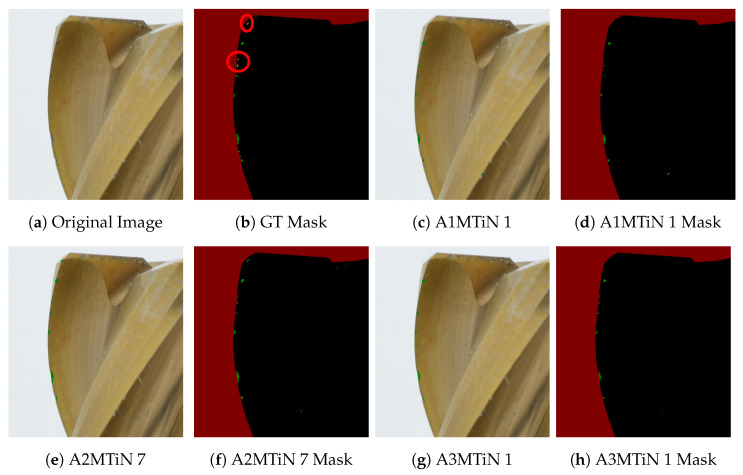
Prediction results and corresponding masks on test images from a TiN-coated milling tool, predicted by the best-performing models of the three annotators, 1, 2 and 3, as detailed and bold in Table A1. The prediction includes the four classes: normal wear in green, abnormal wear in yellow, background in red, and tool in black. Wrong annotations in the GT Mask are marked red.

**Figure 12:**
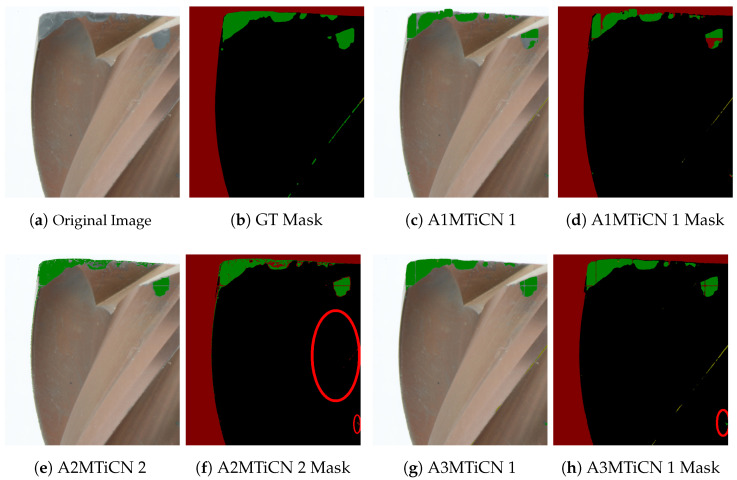
Prediction results and corresponding masks on test images from a TiN-coated milling tool, predicted by the best-performing models of the three annotators, 1, 2 and 3, as detailed and bold in Table A2. The prediction includes the four classes: normal wear in green, abnormal wear in yellow, background in red, and tool in black. Critical regions, such as wrong predictions or missed wear recognition, are marked in red.

The segmentation result of the best-performing model of each annotator is visually shown in Figure 11 for the TiN-coated dataset and in Figure 12 for the TiCN dataset. It can be clearly seen that the reference annotation in the GT in Figure 11b was not correct, and abnormal wear was mistakenly annotated as normal wear, highlighting the relevance of an annotation guideline. For the TiN tool, all models that performed best for the annotators were able to predict wear correctly (Figure 11c,e,g). In contrast for the TiCN tool, in Figure 12c,e,h, it can be seen that abnormal wear was partially predicted as a background. While the acquisition systems reduce reflections, the top of the tool still shows light artifacts, especially in combination with wear. This is because the wear can behave as a scattering source that can appear brighter at a certain angle to the observer, leading to misclassification of wear as a background, as seen in Figure 12c,e,g. This can be improved by increasing the number of training datasets or adjusting the lighting intensity or integrating time of the camera sensor. Furthermore, it can be seen from Figure 12e,f that normal and abnormal wear at the cutting edges remains completely undetected in the A2MTiCN model (marked in red), while the other models of the two annotators detect wear but have difficulties, especially in the subtle transition from normal to abnormal wear. Nevertheless, the results are considered good in quantitative terms.

### 4.5. Coefficient of Variation Analysis of the Segmentation Results across Annotators, Classes, and Hyperparameter Variations

In this section, we present the results of our analysis aimed at validating the inconsistencies in the labeling process and its impact on model performance, which was performed by three annotators with different levels of expertise. The analysis focuses on four different classes: background, tool, normal wear, and abnormal wear. We used the coefficient of variation of mIoU (Equation (5)) to assess the consistency and reliability of the method, as well as evaluate the variation in the readings of individual classes by Annotator 1, Annotator 2, and Annotator 3. Additionally, we investigated the CV associated with different hyperparameters, including DO, LR, and BS, which affected model performance. Our goal was to identify reference points for improving the annotation process and to understand the influence of annotator expertise and model hyperparameters on the quality of dataset annotations.

The following tables (Table 3 for the TiN-coated dataset and Table 4 for the TiCN-coated dataset) present the CV of the IoU values of different models across the different classes, including the wmIoU. The mIoU CV values provide insights into the relative variability of the IoU values for each category across different models. A higher CV indicates greater dispersion around the mean, suggesting that the performance of the models is less consistent in that category.

For the TiN-coated tool, the highest mean CV values are observed in the class “Normal Wear” at 9.48% followed by “Abnormal Wear” at 8.61%, indicating significant variability in model performance in these categories. In contrast, the “Background” class, with a CV value of 0.07%, shows the least variability, followed by the class “Tool” with only 2.75%, suggesting consistent performance across models. It seems that the novel acquisition system enables the model with all hyperparameter combinations to segment the tool from the background effectively, and annotations seem to be performed well by all annotators.

For the TiCN-coated tool, the background has a small mean CV value of 0.76%, but the mean CV value of 9.97% is high compared to the one for the TiN-coated tool, indicating that certain hyperparameters can be beneficial for enhancing the extraction of the tool pixel-wise from the other classes. The mean CV for “Normal Wear” of 20.24% and for the class “Abnormal Wear” of 18.53% exhibits the highest mean CV values, indicating notable variability.

## 5. Conclusions

In this study, we presented an approach to compare the annotation quality and consequent wear detection performance of different CNN models, each trained on datasets created by annotators with varying levels of expertise. The images are derived from TiN- and TiCN-coated milling tools. To achieve this, we utilized a new imaging system designed to minimize reflection and produce high-quality images. Additionally, we analyzed the influence of various hyperparameters to generalize the test datasets and discussed the sensitivity of potentially inconsistent annotations.

The hyperparameters of DO of 0.3 and LR of 0.001 showed consistent model performance in terms of wear detection across all annotations. Annotator 1 achieved a maximum mIoU of 0.8153 for abnormal wear and 0.7120 for normal wear on the TiN datasets. Annotator 3’s models delivered an mIoU of 0.7538 for abnormal wear and 0.5679 for normal wear, with Annotator 2’s performance falling in between these values. The TiCN dataset exhibited a similar trend but with significantly poorer results, indicating annotation challenges due to the subtle wear nature of the tool. This was further demonstrated through the coefficient of variation (CV). The TiN tool showed a low mean CV for overall wmIoU performance at 8.6%, while the TiCN dataset performed significantly worse, with a mean CV of 16.32% for wmIoU.

The results demonstrate the complexity of wear annotation challenges. These findings underscore the importance of professional annotation guidelines, high-resolution images, and large datasets encompassing various types of wear. The three annotators illustrated that specific expertise in machining technology is crucial for the labeling process.

## 6. Patents

The illumination technique used in our research for the wear inspection system is based on the European patent EP1430720, developed by Mühenad Bilal and Christian Mayer. This specific illumination approach has been modified for wear characterization and enables the identification of tiny wear features that cannot be detected with conventional inspection systems.

## Figures and Tables

**Table 1:** Relevant training parameters for the CNN.

Parameters	Value
Image Size	512 × 512 × 3
Image Format	Jpeg
BS	8, 16
DO	0.0, 0.3, 0.5
Epochs	70
GPU’s	1
Trainable Parameters	2,140,740
Loss	Sparse Categorical Cross Entropy
Optimizer	RMS Prop
Metric	IoU
Train/Valid/Test	0.8/0.1/0.1

**Table 2:** Instance distribution across datasets.

Tool Coating	Class Background	Class Normal Wear	Class Abnormal Wear	Class Tool
TiCN	432	404	806	768
TiN	432	770	532	768

**Table 3:** CV for TiN-coated tool.

Model	Background CV [%]	Tool CV [%]	Abnormal Wear CV [%]	Normal Wear CV [%]	wmIoU CV [%]	LR	BS	DO
MTiN 0	0.13	0.24	7.39	9.61	8.91	0.001	8	0
MTiN 1	0.10	2.22	7.49	10.05	8.90	0.001	8	0.3
MTiN 2	0.04	0.37	2.82	10.05	8.07	0.001	8	0.5
MTiN 3	0.18	16.78	9.27	18.49	12.16	0.001	16	0
MTiN 4	0.08	1.69	8.07	2.04	3.12	0.001	16	0.3
MTiN 5	0.03	0.39	11.47	9.94	6.17	0.001	16	0.5
MTiN 6	0.04	2.58	9.92	11.24	10.66	0.0001	8	0
MTiN 7	0.01	1.09	10.02	8.14	7.43	0.0001	8	0.3
MTiN 8	0.03	2.73	7.32	7.42	7.30	0.0001	8	0.5
MTiN 9	0.03	0.81	20.50	17.74	18.45	0.0001	16	0
MTiN 10	0.03	1.41	3.89	4.43	4.26	0.0001	16	0.3
MTiN 11	0.04	1.89	5.41	3.67	3.35	0.0001	16	0.5
Mean CV	0.07	2.75	8.25	9.48	8.61	-	-	-

**Table 4:** CV for TiCN-coated tool.

Model	Background CV [%]	Tool CV [%]	Abnormal Wear CV [%]	Normal Wear CV [%]	wmIoU CV [%]	LR	BS	DO
MTiCN 0	4.69	34.34	41.62	28.19	29.63	0.001	8	0
MTiCN 1	0.31	8.99	12.09	12.93	11.60	0.001	8	0.3
MTiCN 2	0.45	2.16	13.62	14.03	10.32	0.001	8	0.5
MTiCN 3	0.35	10.38	16.86	41.67	30.34	0.001	16	0
MTiCN 4	0.47	6.15	28.05	39.32	27.51	0.001	16	0.3
MTiCN 5	0.54	6.17	16.78	22.67	17.78	0.001	16	0.5
MTiCN 6	0.30	10.26	27.67	33.01	23.48	0.0001	8	0
MTiCN 7	0.31	2.15	4.14	13.69	8.54	0.0001	8	0.3
MTiCN 8	0.42	6.52	15.28	23.34	17.21	0.0001	8	0.5
MTiCN 9	0.26	4.93	28.74	16.33	9.64	0.0001	16	0
MTiCN 10	0.36	16.51	17.60	9.06	7.86	0.0001	16	0.3
MTiCN 11	0.44	2.84	10.02	8.05	5.69	0.0001	16	0.5
Mean CV	0.76	9.97	18.53	20.24	16.32	-	-	-

## Data Availability

The data supporting the findings of this study are owned by WMH Herion Antriebstechnik GmbH and are not publicly available due to proprietary restrictions. However, data may be available from the authors upon reasonable request and with permission of WMH Herion Antriebstechnik GmbH.

## Abbreviations

The following abbreviations are used in this manuscript:

(CNN)	Convolutional Neural Networks
(TiN)	Titanium Nitride
(TiCN)	Titanium Carbonitride
(CV)	Coefficient of Cariation
(DCNN)	Deep Convolutional Neural Networks
(IAS)	Image Acquisition System
(LR)	Learning Rate
(BS)	Batch Size
(DO)	Dropout Rate
(mIoU)	mean Intersection over Union
(wmIoU)	weighted mean Intersection over Union

## Appendix A

**Table A1:** Comparison of the segmentation results as mIoU for the four classes: background, tool, abnormal wear, normal wear, and the overall performance wmIoU using the test dataset of a TiN-coated milling cutter labeled by Annotator 1, Annotator 2, and Annotator 3 with variation of the hyperparameters: LR, DO and BS.

Annotator 1	Background [mIoU]	Tool [mIoU]	Abnormal Wear [mIoU]	Normal Wear [mIoU]	wmIoU [mIoU]	LR	BS	DO
A1MTiN 0	0.9987	0.9895	0.7537	0.6134	0.6472	0.001	8	0
**A1MTiN 1**	0.9986	0.9979	**0.8153**	**0.7120**	**0.7369**	0.001	8	0.3
A1MTiN 2	0.9980	0.9890	0.6866	0.7120	0.7067	0.001	8	0.5
A1MTiN 3	0.9983	0.9459	0.6361	0.7120	0.6947	0.001	16	0
A1MTiN 4	0.9988	0.9982	0.8105	0.5935	0.6453	0.001	16	0.3
A1MTiN 5	0.9978	0.9962	0.5122	0.7039	0.6596	0.001	16	0.5
A1MTiN 6	0.9986	0.9410	0.6686	0.3597	0.4335	0.0001	8	0
A1MTiN 7	0.9978	0.9724	0.6243	0.5875	0.5970	0.0001	8	0.3
A1MTiN 8	0.9979	0.9888	0.6623	0.6619	0.6627	0.0001	8	0.5
A1MTiN 9	0.9986	0.9816	0.7119	0.4792	0.5350	0.0001	16	0
A1MTiN 10	0.9981	0.9800	0.6458	0.5161	0.5476	0.0001	16	0.3
A1MTiN 11	0.9981	0.9640	0.5671	0.4312	0.4643	0.0001	16	0.5
**Annotator 2**	**Background [mIoU]**	**Tool [mIoU]**	**Abnormal Wear [mIoU]**	**Normal Wear [mIoU]**	**wmIoU [mIoU]**	**LR**	**BS**	**DO**
A2MTiN 0	0.9958	0.9845	0.6288	0.4853	0.5201	0.001	8	0
A2MTiN 1	0.9964	0.9518	0.6870	**0.5933**	0.6148	0.001	8	0.3
A2MTiN 2	0.9970	0.9969	0.6794	0.5933	0.6144	0.001	8	0.5
A2MTiN 3	0.9945	0.6453	0.5893	0.5933	0.5926	0.001	16	0
A2MTiN 4	0.9970	0.9626	0.6780	0.5933	0.6141	0.001	16	0.3
A2MTiN 5	0.9971	0.9969	0.6780	0.5933	0.6141	0.001	16	0.5
A2MTiN 6	0.9977	0.9980	**0.8082**	0.4618	0.5443	0.0001	8	0
**A2MTiN 7**	0.9980	0.9980	0.7778	0.5881	**0.6335**	0.0001	8	0.3
A2MTiN 8	0.9972	0.9386	0.6073	0.5775	0.5853	0.0001	8	0.5
A2MTiN 9	0.9979	0.9818	0.7234	0.4504	0.5157	0.0001	16	0
A2MTiN 10	0.9973	0.9647	0.5908	0.4646	0.4954	0.0001	16	0.3
A2MTiN 11	0.9972	0.9717	0.5334	0.3949	0.4287	0.0001	16	0.5
**Annotator 3**	**Background [mIoU]**	**Tool [mIoU]**	**Abnormal Wear [mIoU]**	**Normal Wear [mIoU]**	**wmIoU [mIoU]**	**LR**	**BS**	**DO**
A3MTiN 0	0.9982	0.9895	0.7047	0.5400	0.5797	0.001	8	0
**A3MTiN 1**	0.9985	0.9981	**0.7538**	**0.5679**	**0.6125**	0.001	8	0.3
A3MTiN 2	0.9974	0.9968	0.6435	0.5679	0.5866	0.001	8	0.5
A3MTiN 3	0.9983	0.9470	0.7347	0.4478	0.5163	0.001	16	0
A3MTiN 4	0.9983	0.9976	0.6954	0.5681	0.5990	0.001	16	0.3
A3MTiN 5	0.9976	0.9883	0.5852	0.5599	0.5668	0.001	16	0.5
A3MTiN 6	0.9983	0.9899	0.6508	0.3757	0.4417	0.0001	8	0
A3MTiN 7	0.9981	0.9803	0.6432	0.4918	0.5285	0.0001	8	0.3
A3MTiN 8	0.9978	0.9299	0.5534	0.5599	0.5592	0.0001	8	0.5
A3MTiN 9	0.9983	0.9649	0.4452	0.3112	0.3442	0.0001	16	0
A3MTiN 10	0.9980	0.9467	0.6381	0.5037	0.5363	0.0001	16	0.3
A3MTiN 11	0.9977	0.9302	0.4966	0.4207	0.4397	0.0001	16	0.5

**Table A2:** Comparison of the segmentation results as mIoU for the four classes: background, tool, abnormal wear, normal wear, and the overall performance wmIoU using the test dataset of a TiCN-coated milling cutter labeled by Annotator 1, Annotator 2, and Annotator 3 with variation of the hyperparameters: LR, DO and BS.

Annotator 1	Background [mIoU]	Tool [mIoU]	Abnormal Wear [mIoU]	Normal Wear [mIoU]	wmIoU [mIoU]	LR	BS	DO
A1MTiCN 0	0.9961	0.8439	0.5229	0.4586	0.5305	0.001	8	0
**A1MTiCN 1**	0.9964	0.9548	**0.6568**	0.5847	**0.6536**	0.001	8	0.3
A1MTiCN 2	0.9958	0.9262	0.4596	0.5072	0.5842	0.001	8	0.5
A1MTiCN 3	0.9969	0.9551	0.6136	0.5445	0.6209	0.001	16	0
A1MTiCN 4	0.9959	0.8173	0.2934	0.2174	0.3290	0.001	16	0.3
A1MTiCN 5	0.9955	0.8322	0.5638	**0.5934**	0.6375	0.001	16	0.5
A1MTiCN 6	0.9975	0.8219	0.4585	0.4561	0.5239	0.0001	8	0
A1MTiCN 7	0.9975	0.8984	0.5759	0.4794	0.5576	0.0001	8	0.3
A1MTiCN 8	0.9966	0.8050	0.4100	0.4402	0.5076	0.0001	8	0.5
A1MTiCN 9	0.9971	0.7031	0.3886	0.3675	0.4301	0.0001	16	0
A1MTiCN 10	0.9964	0.5983	0.3486	0.3672	0.4104	0.0001	16	0.3
A1MTiCN 11	0.9963	0.7507	0.3119	0.2970	0.3812	0.0001	16	0.5
**Annotator 2**	**Background [mIoU]**	**Tool [mIoU]**	**Abnormal Wear [mIoU]**	**Normal Wear [mIoU]**	**wmIoU [mIoU]**	**LR**	**BS**	**DO**
A2MTiCN 0	0.8983	0.3533	0.1599	0.2571	0.2750	0.001	8	0
A2MTiCN 1	0.9961	0.7911	0.4870	0.4249	0.4933	0.001	8	0.3
**A2MTiCN 2**	0.9951	0.9407	**0.5947**	**0.5617**	0.6320	0.001	8	0.5
A2MTiCN 3	0.9962	0.7667	0.4076	0.1881	0.2970	0.001	16	0
A2MTiCN 4	0.9970	0.9378	0.5878	0.5555	0.6264	0.001	16	0.3
A2MTiCN 5	0.9958	0.9005	0.3705	0.5934	**0.6485**	0.001	16	0.5
A2MTiCN 6	0.9955	0.7428	0.3519	0.1957	0.2984	0.0001	8	0
A2MTiCN 7	0.9974	0.9396	0.5853	0.3547	0.4646	0.0001	8	0.3
A2MTiCN 8	0.9970	0.8960	0.5699	0.4499	0.5333	0.0001	8	0.5
A2MTiCN 9	0.9968	0.7905	0.4986	0.2600	0.3601	0.0001	16	0
A2MTiCN 10	0.9959	0.9018	0.5048	0.3617	0.4627	0.0001	16	0.3
A2MTiCN 11	0.9965	0.8019	0.3385	0.3434	0.4283	0.0001	16	0.5
**Annotator 3**	**Background [mIoU]**	**Tool [mIoU]**	**Abnormal Wear [mIoU]**	**Normal Wear [mIoU]**	**wmIoU [mIoU]**	**LR**	**BS**	**DO**
A3MTiCN 0	0.9915	0.8627	0.4124	**0.5363**	0.5957	0.001	8	0
**A3MTiCN 1**	0.9896	0.9716	0.5922	0.5314	**0.6132**	0.001	8	0.3
A3MTiCN 2	0.9859	0.8933	**0.6410**	0.3969	0.4906	0.001	8	0.5
A3MTiCN 3	0.9893	0.7773	0.4876	0.3245	0.4097	0.001	16	0
A3MTiCN 4	0.9866	0.8350	0.5773	0.3129	0.4116	0.001	16	0.3
A3MTiCN 5	0.9843	0.7744	0.4834	0.3475	0.4277	0.001	16	0.5
A3MTiCN 6	0.9904	0.6380	0.2249	0.3151	0.3746	0.0001	8	0
A3MTiCN 7	0.9909	0.8976	0.6325	0.4854	0.5628	0.0001	8	0.3
A3MTiCN 8	0.9880	0.7684	0.4272	0.2561	0.3523	0.0001	8	0.5
A3MTiCN 9	0.9914	0.7691	0.2361	0.3842	0.4545	0.0001	16	0
A3MTiCN 10	0.9886	0.7510	0.5341	0.4391	0.4978	0.0001	16	0.3
A3MTiCN 11	0.9870	0.7921	0.2646	0.2860	0.3796	0.0001	16	0.5
